# Revascularization of an Autotransplanted Mature Tooth After Extraoral Root Resection: A Case Report

**DOI:** 10.1155/crid/5545344

**Published:** 2025-01-17

**Authors:** Juraj Marton, Michal Mozoľa, Radovan Žižka, Zdeněk Pokorný

**Affiliations:** ^1^Institute of Dentistry and Oral Sciences, Faculty of Medicine, Palacky University, Olomouc, Czech Republic; ^2^Department of Oral and Maxillofacial Surgery, Faculty Hospital, Olomouc, Czech Republic

**Keywords:** case report, mature tooth, pulse oximetry, revascularization, tooth autotransplantation

## Abstract

The outcome of tooth autotransplantation depends mainly on the transplant tooth's anatomy—the type of donor tooth and the developmental stage of root formation. Mature teeth display a higher complication rate due to lower pulp revascularization potential, requiring root canal treatment (RCT) pre- or postoperatively to avoid postoperative complications, which extends treatment duration and cost. This report details a 39-year-old patient's autotransplantation of a mature wisdom tooth to replace the first molar after unsuccessful root canal retreatment. During the surgery, an extraoral root resection of the transplanted tooth was performed prior to placement to avoid the need to elevate the Schneiderian membrane, which displayed imperfect healing following the surgical removal of a cystic lesion in the maxillary sinus. RCT was not performed before nor after the procedure. At the 3-year follow-up, the tooth was asymptomatic. The vitality of the autotransplanted tooth was difficult to determine using standard vitality tests, which depend on patients' subjective responses, but the use of pulse oximetry objectively confirmed this. This case not only shows the possibility of a mature tooth transplant revascularization in an older patient but also gives a possible postoperative protocol of how to objectively confirm and measure the revascularization of the autotransplanted tooth.

## 1. Introduction

In tooth autotransplantation, mature teeth pose a greater surgical risk due to complex root anatomy, increasing the risk of damage to the periodontal ligaments (PDLs) during extraction and the reduced likelihood of pulp revascularisation, thus necessitating root canal treatment (RCT) [1]. In recent years, several authors explored the potential of extraoral root resection (EORR) as a means to reduce the necessity for RCT. Case reports have demonstrated the potential benefits of this approach in fully developed premolars, particularly in younger patients with follow-ups ranging from 18 months [2] to 3 years [3]. Furthermore, a retrospective study published in 2020 investigated the outcomes of nine mature teeth, including canines, premolars, and molars undergoing apicoectomy during autotransplantation, with only one failure necessitating RCT; the study suggested promising outcomes, especially for single-rooted transplants [4]. Notably, all patients in this study were under 30 years of age.

In the following case, we present successful autotransplantation of an impacted mature wisdom tooth in a 39-year-old patient. EORR was performed to avoid the need for elevating the Schneiderian membrane in the recipient socket, considering the patient's history of extensive surgical removal of a cystic lesion in the maxillary left sinus. Following the surgery, RCT was not performed. At the 3-year follow-up, the tooth showed no clinical or radiological pathologies. To objectively measure the tooth's vitality, pulse oximetry was used alongside the patient's subjective responses, that is, the electric pulp test (EPT) and cold sensitivity test.

## 2. Materials and Methods

This case is reported according to the 2013 CAse REport (CARE) Checklist.

### 2.1. Primary Situation and Treatment Planning

**Figure 1:**
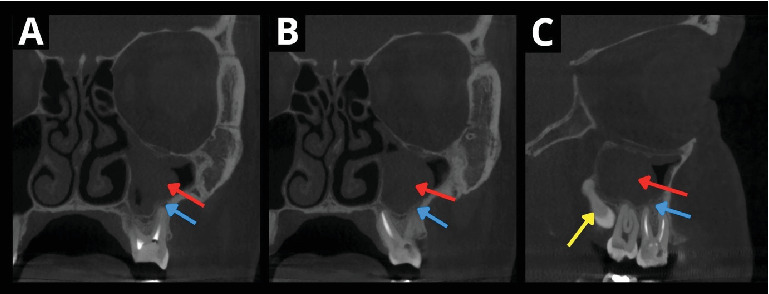
Preoperative x-ray examination. The coronal section CBCT reveals an oval opacity in the left maxillary sinus (A–C) (red arrow). The compact bone remains intact (blue arrow). The lesion exhibits characteristics of a mucocele. The parasagittal section CBCT shows an impacted wisdom tooth with a single conical root in a horizontal position (C) (yellow arrow). The tooth appears to be the optimal donor for autotransplantation.

A 39-year-old female was referred by her general dental practitioner to the Department of Oral and Maxillofacial Surgery at Faculty Hospital Olomouc in January 2019 for an extraction of the upper left first molar (Tooth #14) due to the failed root canal retreatment and an impacted upper left wisdom tooth (Tooth #16). The patient was treated at otorhinolaryngology (ENT) for chronic symptoms of pain localized to the left maxilla, where an x-ray examination revealed an oval opacity in the left maxillary sinus (Figures 1(A) and 1(B)). Aside from the ENT treatment, the patient had no significant medical history, was in good health, did not take any long-term medication, and had no allergies.

The clinical examination showed an impacted upper left wisdom tooth. Tooth #14 had a suspected poor-quality composite restoration with a visible defect. It was sensitive to percussion and had a periodontal probing depth within normal limits. The x-ray examination showed inadequate RCT and suspected periapical pathology of the mesiobuccal root. The tooth was diagnosed with chronic apical periodontitis. To examine the impacted maxillary molar more accurately, a cone beam CT (CBCT) was conducted. The CBCT examination revealed a wisdom tooth with one conical root, composed of fused roots, and one wide “C” shaped root canal (class one according to Melton, Krell, and Fuller [5]), impacted behind the second molar in a horizontal position (Figure 1(C)). Hyperplasia of the left sinus mucosa was observed, and the ostium was open. The left maxillary sinus lesion was initially diagnosed as a mucocele due to the intact compact bone and the mucosal characteristics, which were later confirmed histologically.

**Figure 2:**
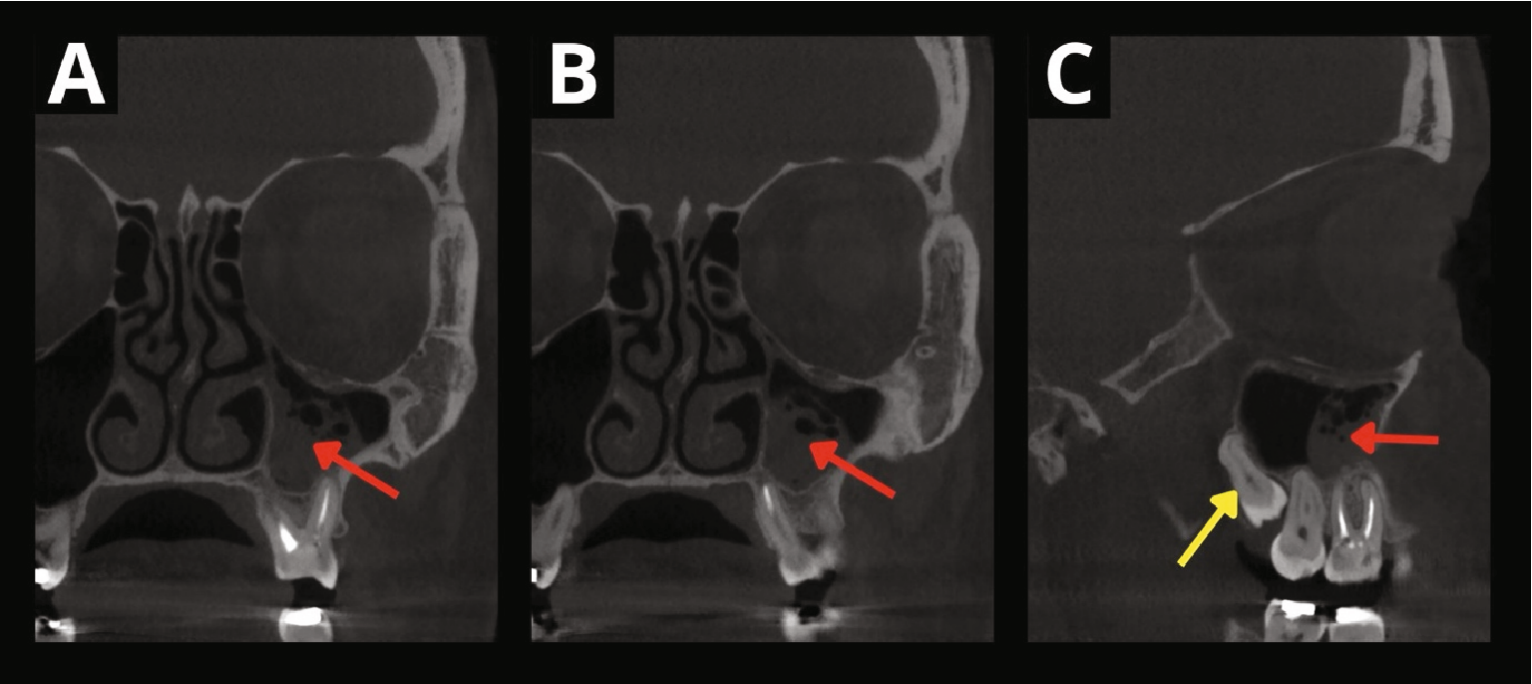
X-ray examination after the removal of the cystic lesion. The lesion was histologically diagnosed as a mucocele. The CBCT reveals incomplete healing of the sinus mucosa (A–C) (red arrow). The preoperative position of the donor tooth can be observed (C) (yellow arrow).

The patient was offered an autotransplantation of the wisdom tooth to replace the first molar after the removal of the cystic lesion from the maxillary sinus. The cystic lesion filled up with mucus and pus was removed in May 2019 endoscopically, and the patient underwent antibiotic therapy (Table 1). In September 2019, the patient came back for a preoperative check-up before autotransplantation. The CBCT scan showed incomplete healing of the mucosa in the maxillary sinus (Figures 2(A), 2(B), and 2(C)). The treatment plan was thoroughly explained once again during the preoperative examination. The patient agreed to the proposed procedure and signed the informed consent form.

### 2.2. Surgical Procedure

A month after the preoperative check-up, the maxillary left wisdom tooth was immediately autotransplanted to replace the maxillary left first molar. The patient was instructed to take 1 g of amoxicillin (Amoksiklav, Sandoz, Switzerland) 1 h before the surgery.

Retromaxillary tuber anaesthesia was administered using 4% articaine (Supracain, Zentiva, Prague, Czech Republic), and the left upper first molar was surgically extracted after sectioning the roots. The recipient socket was prepared by removing the inter-radicular septum using a round bur in a low-speed handpiece. No additional preparation was performed to preserve bone and minimize the risk of oro-antral communication.

Subsequently, the full-thickness flap was raised in the region of Tooth #16, and a conservative osteotomy over the crown of the wisdom tooth was performed; the upper left wisdom tooth was then atraumatically extracted with meticulous care using minimal use of elevators to reduce any damage to PDL.

Afterwards, approximately 4 mm of the root length (the apical hook) was resected extraorally with a surgical fissure bur (surgical cutter, Meisinger, Neuss, Germany) to avoid irritation and elevation of the Schneiderian membrane.

Subsequently, the donor tooth (#16) was carefully placed in the recipient socket and secured with two cross-mattress sutures attached to the crown via flowable composite. To minimize interference, the transplanted tooth was kept out of occlusion.

### 2.3. Postoperative Care

The patient was instructed not to brush around the surgical wound, avoid rinsing for a few days, apply a cold apparatus to the surgical site, and avoid hard food for 2 weeks. The prescribed postoperative medication was 1 g of amoxicillin (Amoksiklav, Sandoz, Basel, Switzerland) every 12 h for 7 days. If analgesics were needed, 100 mg of nimesulide (Aulin, Angelini Pharma, Brno, Czech Republic) was prescribed every 12 h. Probiotics were recommended.

## 3. Results

**Figure 3:**
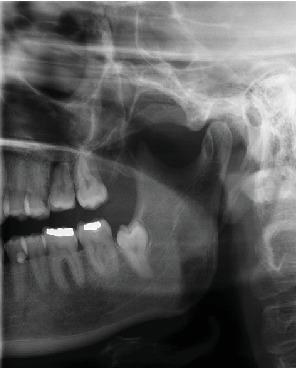
Postoperative x-ray examination. The orthopantomogram (OPG) shows the position of the transplanted tooth 13 days after surgery.

The follow-up check-ups were conducted at 3, 13, and 20 days postoperatively (Table 1). A panoramic x-ray (Figure 3) was performed 13 days postsurgery to assess the position of the transplant. The sutures were removed following the extraction, whilst the tooth fixation remained in place. There were no signs of pathology observed 20 days after the transplantation. Subsequently, 2 weeks later, the surgeon proceeded to remove the fixation. The elective RCT was originally planned during the removal of fixation but could not be performed because of the COVID-19 pandemic.

Due to the pandemic, Olomouc University Hospital's care was reduced to acute conditions only. Therefore, the patient was informed about potential risks and that an RCT would be initiated only in case of complication according to the Pogrel protocol [6]. Later that year, the patient contacted the department and informed the surgeon about experiencing a sensation in the tooth and feeling a normal response to cold stimuli.

**Figure 4:**
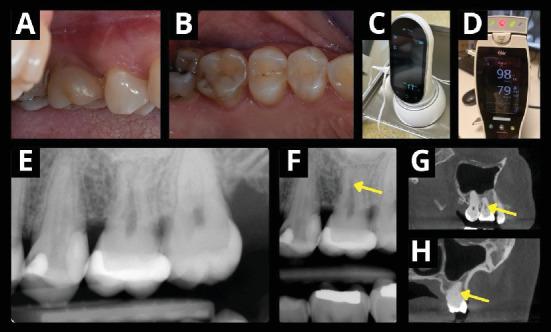
Three-year follow-up. (A, B) The tooth shows no clinical signs of pathologies. The vitality was assessed using (C) electric pulp test (Woodpecker Ai-Pex, Guilin Woodpecker Medical Instruments Co., Guilin, Guangxi, China) and (D) pulse oximetry (Radical-7 Pulse CO-Oximeter, Masimo, Irvine, California, United States). (E) The x-ray examination reveals signs of hard tissue apposition in the pulp chamber without periapical lesion or root resorption (F–H) (yellow arrow) compared to the 1-year follow-up.

Three years after the transplantation, the transplanted tooth exhibited no clinical signs of pulp pathologies (Figures 4(A) and 4(B)). It demonstrated a slight delay in response compared to the contralateral upper first molar (#3) during a cold sensitivity pulp test. The results were verified by an EPT (Woodpecker Ai-Pex, Guilin Woodpecker Medical Instruments Co., Guilin, Guangxi, China), giving similar outcomes. The intraoral x-ray (Figure 4(F)) and CBCT (Figures 4(G) and 4(H)) of the transplanted tooth showed evidence of possible hard tissue apposition in the pulp chamber compared to the 1-year follow-up x-ray (Figure 4(E)).

To objectively measure the revascularization of the tooth, the blood saturation in the transplanted tooth was measured using a modified pediatric pulse oximeter (Radical-7 Pulse CO-Oximeter, Masimo, Irvine, California, United States) with a neonatal foot probe wrapped around the tooth as previously described [7, 8] (Figure 4(D)). The comparison of all measured values between the autotransplanted tooth and the control tooth is presented in Table 2.

The patient was satisfied with the outcome and was gladly willing to show up for a follow-up and cooperated during postoperative tests to evaluate the outcomes. She gave consent for the publication.

## 4. Discussion

Pulp necrosis, alongside the damage of PDL, leads to external inflammatory root resorption, which is one of the leading causes of autotransplanted tooth failure. The likelihood of necrosis following replantation or autotransplantation increases with the critical size of the defect—smaller apical diameters and higher pulp lengths exacerbate the risk. According to Andreasen et al., optimal conditions for predictable revascularization include a minimal apical diameter of 1 mm and a maximal pulp length of 14 mm [1]. Histological studies provide inconsistent findings regarding the relationship between pulp revascularization, apical diameter, and pulp length, as they demonstrate new tissue ingrowth even in cases with smaller apical diameter [9]. Clinical studies show a higher potential for revascularization in cases with incomplete root formation or a wider apical diameter [10]. Interestingly, the presence of uninfected necrotic tissue in the pulp chamber appears to stimulate new tissue ingrowth [11].

RCT protocols for mature teeth undergoing autotransplantation typically involve initiating RCT 2–4 weeks after surgery [12] to preserve periodontal healing. Alternatively, RCT may be conducted before surgery if tooth access is feasible, aiming to reduce the likelihood of postoperative complications. Additionally, some authors recommend performing extraoral apicoectomy along with retrograde filling using mineral trioxide aggregate (MTA) [13, 14]. An alternative approach is to consider elective RCT only upon the emergence of initial signs of pathology, whether clinical or radiological [6, 15].

Several cases and a retrospective study have explored the use of EORR during autotransplantation to avoid RCT. Case reports involving fully developed premolars showed successful outcomes with follow-ups ranging from 18 months [2] up to 3 years [3], all in young patients. In a 2020 retrospective study, nine mature teeth (three canines, two premolars, and four molars) underwent apicoectomy during autotransplantation, with only one failure (second mandibular molar) necessitating RCT [4]. Notably, single-rooted transplants showed promising results, with four out of nine teeth exhibiting obliteration on x-ray. Success assessment in these cases relied on x-ray examination and EPT or cold sensitivity test, although diagnosis posed challenges due to the subjective responses in patients, all of whom were under 30 years old. This approach carries the risk of challenges in assessing pulp revascularization postsurgery.

The challenge of vitality assessment after autotransplantation comes from the lack of objectivity in the used tests, that is, the cold sensitivity test and EPT. Both tests depend on the patient's subjective response to the stimuli. An alternative approach may present pulse oximetry with the use of modified probes, giving objectively measurable values of the pulp tissue saturation that do not depend on the patient's response [7, 8].

In our report, a successful autotransplantation of a fully developed wisdom tooth with EORR was performed in an older (39-year-old) patient. At the 3-year follow-up, the tooth remained asymptomatic, with radiographic evidence showing hard tissue apposition in the pulpal chamber and no periapical lesion. Notably, the tooth exhibited a positive response to a cold sensitivity test and EPT. Objective confirmation of pulp revascularization was obtained through pulse oximetry, offering quantifiable values and addressing the challenge of determining vitality posttransplantation.

## 5. Conclusions

To minimize the risk of pulp necrosis of the autotransplanted tooth, EORR seems to be the optimal method to increase pulp revascularization by widening the apical diameter and shortening the pulp length. The revascularization after surgery can be objectively evaluated using pulse oximetry. To establish guidelines and adopt this technique as a standard for mature tooth autotransplantation, prospective studies with a well-defined surgical protocol and a homogenous donor group are needed.

## Figures and Tables

**Table 1:** The timeline.

**Date**	**Procedure**
24.11.2017	Patient's first visit, referred for extraction of Teeth #14 and #16, indication for a CBCT scan
29.11.2017	CBCT scan reading, hyperplasia in maxillary sinus detected, periapical lesion Tooth #14, the patient reffered to evaluate the possibility of root canal retreatment (RCRT)
29.1.2019	The patient was again referred for extraction of Tooth #14 after failed RCRT and Tooth #16, CBCT scan indicated
1.2.2019	CBCT scan reading, the patient referred to ENT for evaluation of maxillary sinus lesion
2.5.2019	ENT diagnosed the lesion as a mucocele
24.5.2019	Endoscopic removal of cystic lesion in the maxillary sinus
30.5.2019	Postoperative check-up, antibiotic therapy according to the cultivation (*Staphylococcus epidermidis*)
4.9.2019	Check-up before tooth autotransplantation, preoperative CBCT revealed complicated maxillary sinus healing
4.10.2019	Tooth autotransplantation
7.10.2019	Postoperative check-up, intraoral x-ray
17.10.2019	Postoperative check-up
31.10.2019	Postoperative check-up
3.5.2022	Check-up, positive vitality check (cold test, electric pulp test, and oximetry), CBCT scan
7.3.2023	Most recent check-up, vitality check, intraoral x-ray

**Table 2:** The comparison of the autotransplanted tooth to the control tooth.

	**Tooth #14 (transplant)**	**Tooth #3 (control)**
Cold sensitivity (VAS; presence of delay in reaction)	2; 5 s	2; 1 s
EPT (positive value)	17	12
Pulse oximetry (saturation)	98%	98%

## Data Availability

The data that support the findings of this study are available from the corresponding author upon reasonable request.
